# Minimally invasive sacroiliac fusion, a case series, and a literature review

**DOI:** 10.1051/sicotj/2022042

**Published:** 2022-10-25

**Authors:** Mohammad H. Amer, Walid A. Elnahal, Sherif A. Khaled, Khaled F.M. Abdel-Kader, Michael A. Cass, James Gibbs, Philip M. Stott

**Affiliations:** 1 Department of Trauma and Orthopaedics, University Hospital of Sussex NHS Trust, Royal Sussex County Hospital Eastern Road Brighton BN2 5BE United Kingdom; 2 Department of Trauma and Orthopaedics, Cairo University, Al Kasr Al Aini Hospital Old Cairo Cairo 4240310 Egypt; 3 Orthopaedics Department, Armed Forces College of Medicine Cairo 4460015 Egypt; 4 Consultant Spinal Surgeon Spring Orthopaedic Group; Honorary Consultant Spinal Surgeon University Hospitals of Sussex NHS Trust, Montefiore Hospital Montefiore Road Hove BN3 1RD United Kingdom

**Keywords:** Sacroiliac, Fusion, Stabilization, iFuse^®^, Minimally invasive, Sacroiliac dysfunction, Sacroiliitis

## Abstract

*Introduction:* Non-autoimmune sacroiliac joint pain contributes to nearly a quarter of low back pain patients. Non-surgical management fails to satisfy patients. A new minimally invasive technique for sacroiliac stabilization has been introduced, defying the traditional rules of fusion. The results outside explanatory trials and in day-to-day practice have not been reported. *Materials and methods:* This case series includes 20 patients diagnosed with chronic sacroiliac pain resistant to conservative management for at least 6 months. The diagnosis was confirmed with a positive sacroiliac injection. Patients underwent stabilization using the iFuse^®^ implant. Patients were followed up for a minimum of one year. The primary outcome was the functional outcomes, assessed using VAS, ODI, and SF36. Secondary procedure rates, complication rates, and radiological assessments of fusion were collected as secondary outcomes. *Results:* At one year, the mean VAS score improved from 81.25 ± 10.7 SD preoperatively to 52.5 ± 26.8, *p*-value 0.0013. The mean ODI improved from 54.8 ± 11.21 SD preoperatively to 41.315 ± 15.34, *P* value = 0.0079. The mean PCS and MCS of SF36 improved by 17 and 20 points, respectively. Only 55% of patients achieved the MCID for the VAS score. 35% of the cohort had secondary procedures. *Discussion:* Minimally invasive sacroiliac fusion resulted in an improvement in mean functional scores with a wide dispersion. Patients not achieving MCID are patients with either a malpositioned implant, an associated lumbar pathology, or an inaccurate diagnosis. Our results are underwhelming compared to similar work but are still better than conservative cohorts in comparative studies. *Conclusion:* Minimally invasive sacroiliac fusion can be used successfully in select patients. Attention to diagnosis and surgical technique can improve the reproducibility of results.

## Introduction

Low back pain (LBP) is the leading worldwide cause of years lost to disability [1]. The sacroiliac joint (SIJ) plays a role in 15–30% of these patients and even a higher percentage in patients who have had previous lumbar fusion [2]. The effect of SIJ dysfunction on the quality of life is comparable to other well-recognized preoperative Orthopaedic conditions such as lumbar spinal stenosis and degenerative hip arthritis [3].

Conservative management is the first line of treatment. It is not effective in the long run [4, 5], failing to achieve the Minimal Clinical Important Difference (MCID) in 75–90% of patients, and is associated with increased use of opioids [2, 6, 7].

Fusion is the surgical procedure of choice in resistant cases. Minimally invasive (MI) SIJ fusion has been shown to have lower operative blood loss, hospital stay, surgical time, and better pain relief compared to open SIJ fusion [8, 9]. This low morbidity intervention led to a reignited interest in this often forgotten joint with early promising results [2, 6] (Tables 1–3).

A meta-analysis evaluating the quality of evidence in fourteen publications on a commercially available system rated the available evidence as poor [10]. Shamrock et al. reported in a systematic review that seven articles received industry- sponsored funding, and eight articles had at least one author with a relevant disclosure [11].

To our knowledge, the results of this procedure achieved within the national health services (NHS) settings have not been reported, and the question of whether the published results are reproducible outside explanatory trials is yet to be answered.

The primary aim of the study is to report on functional outcomes following MI SIJ fusion, adding to the available literature. Secondary outcomes include complication rate, secondary procedure rate, and a radiological assessment of fusion would highlight challenges with diagnosis, surgical technique, and follow-up.

## Materials and methods

We reviewed the treatment outcomes of 20 consecutive patients treated at a single NHS trust and a local private hospital between January 2019 and March 2021. The mean follow-up was 19.9 ± 3.4 standard deviation (SD) months with a minimum follow-up of 14 months.

Patients were referred with chronic low back pain resistant to conservative management for at least six months. The mean age at the time of surgery was 49.6 ± 14.5 SD years. Fifteen patients (75%) were females. Seven patients (35%) had previous lumbar surgery, six patients (30%) had a history of hip pathology or replacement, and two patients (10%) previously had a contralateral SIJ fusion (Table 2).

Patients were diagnosed as either dynamic sacroiliac dysfunction or degenerative sacroiliitis using history, physical examination, and a positive SI block. The main symptom was pain localizing to SIJ using the Fortin finger test [12]. An examination of SI, hip, and spine, in addition to a limb length assessment was performed in all patients. SI provocative tests were used [13]. X-rays of the pelvis and lumbar spine were performed in all patients to rule out a primary hip or spine cause. An MRI of the lumbar spine was obtained if felt clinically needed.

When symptoms, examination, and imaging pointed towards the SIJ, patients were sent for a diagnostic computed tomography (CT) guided SI injection. A 50% reduction in visual analog score (VAS) right after the procedure was considered the threshold to confirm the SIJ as the primary pain generator [6].

Conservative management was maximized, which included personalized physiotherapy, multiple SI injections, and radiofrequency ablation. Patients with a VAS score of more than 50, an oswestry disability index (ODI) of more than 30, and a positive response to injection failing conservative management were offered MI SI fusion using the iFuse^®^ implant (SI Bone, California USA) [6].

The iFuse^®^ is a triangular-shaped titanium implant with a porous plasma spray coating. It relies on interference fit for initial stability and has a large surface area to promote bone ingrowth to create bony bridges across the SI joint [14].

The procedures were performed by three consultants with expertise in sacroiliac screw placement. Under a general anesthetic with the patient in a supine position on a radiolucent table, a 3 cm lateral skin incision superimposed on the lateral sacral projection is performed. This is followed by a blunt division of gluteal fascia and muscles to reach the outer table of the ilium. Three guide wires were inserted under image guidance in the inlet, outlet, and lateral view corridor across the sacroiliac joint at the level of the S1 body, S1 foramen, and S2 body, respectively. The first wire is placed using a freehand technique as described for iliosacral screw placement [15]. Subsequent wires are inserted using a pin guide system utilizing the first wire as a reference. This is followed by drilling, broaching, and insertion of implant through a soft tissue protection sleeve. The incision is irrigated and closed in layers using absorbable stitches [14, 16]. Patients were kept partial weight bearing for 6 weeks. Follow-up scheduled at 6 weeks and 1 year.

### Primary outcome measure

The pain was assessed using VAS, functional outcome using ODI, and quality of life using short form 36 (SF36) [17, 18]. The scores were collected preoperatively and at 1 year. The MCID for VAS and ODI was set at a reduction of 20 points and 15%, respectively [17, 19].

### Secondary outcome measures

**Figure 1:**
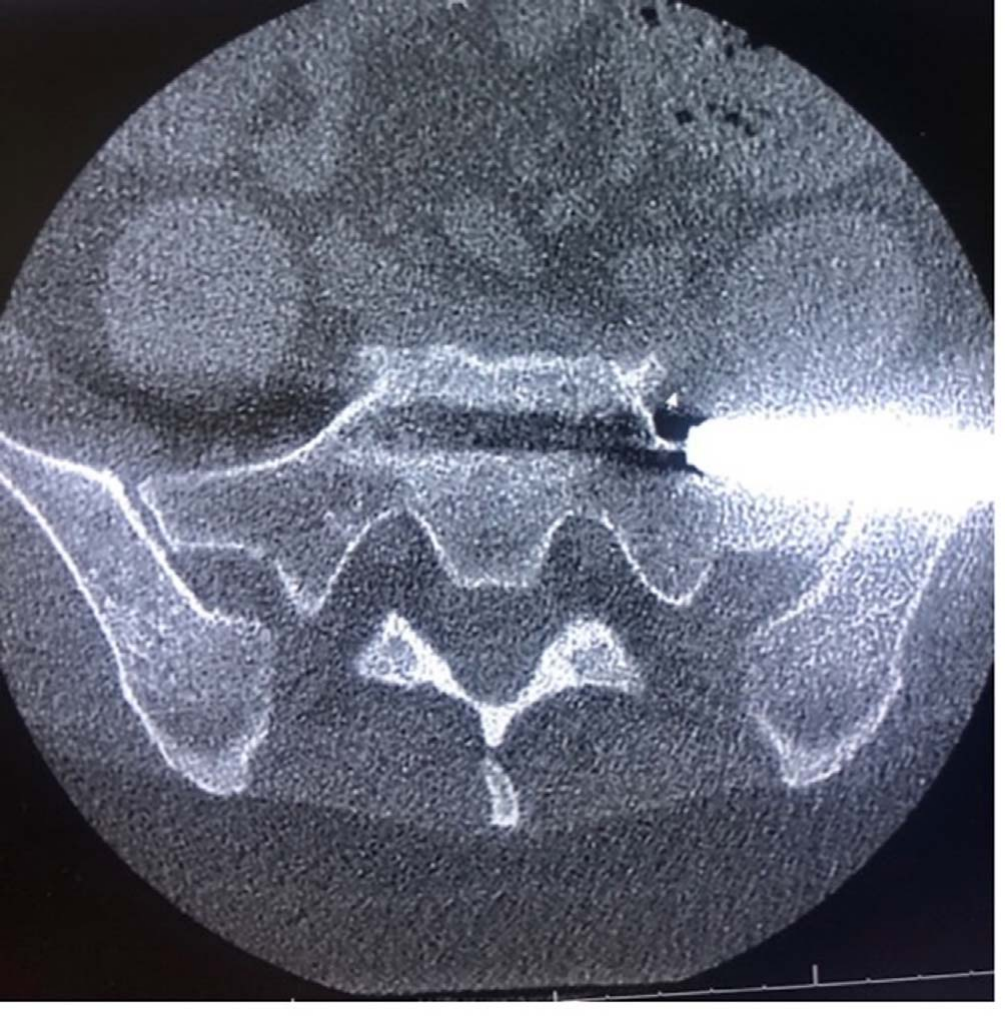
Axial cuts of a CT scan showing sacrum and both sacroiliac joints, note the implant breaches the left sacroiliac corridor anteriorly.

Complications and secondary procedures were recorded up until the final follow-up. Radiological assessment using a pelvis AP X-ray obtained at 1 year and a CT scan when clinically indicated (Figure 1).

Data were coded and entered using graph pad prism version 9. Data were summarized using mean and standard deviation (SD) and using frequency (count) and relative frequency (percentage) for categorical data. Comparisons between quantitative variables were made using the paired student *T*-tests. *p*-Values less than 0.05 were considered statistically significant.

## Results

An overall improvement in pain and functional outcome was observed. The VAS score improved from a mean of 81.25 ± 10.7 preoperatively to 52.5 ± 26.8 at one year, *p*-value of 0.0013. The mean ODI improved from 54.8 ± 11.21 SD preoperatively to 41.315 ± 15.34, *p-*Value = 0.0079. However, only eleven patients (55%) and 10 patients (50%) achieved the MCID for VAS and ODI, respectively.

The quality of life assessed by SF36 improved at 1 year. The mean physical component section (PCS) of SF36 improved from 28.9 preoperatively to 45.6, and the mean mental component section (MCS) changed from 35.3 to 55.85 at one year.

**Figure 2:**
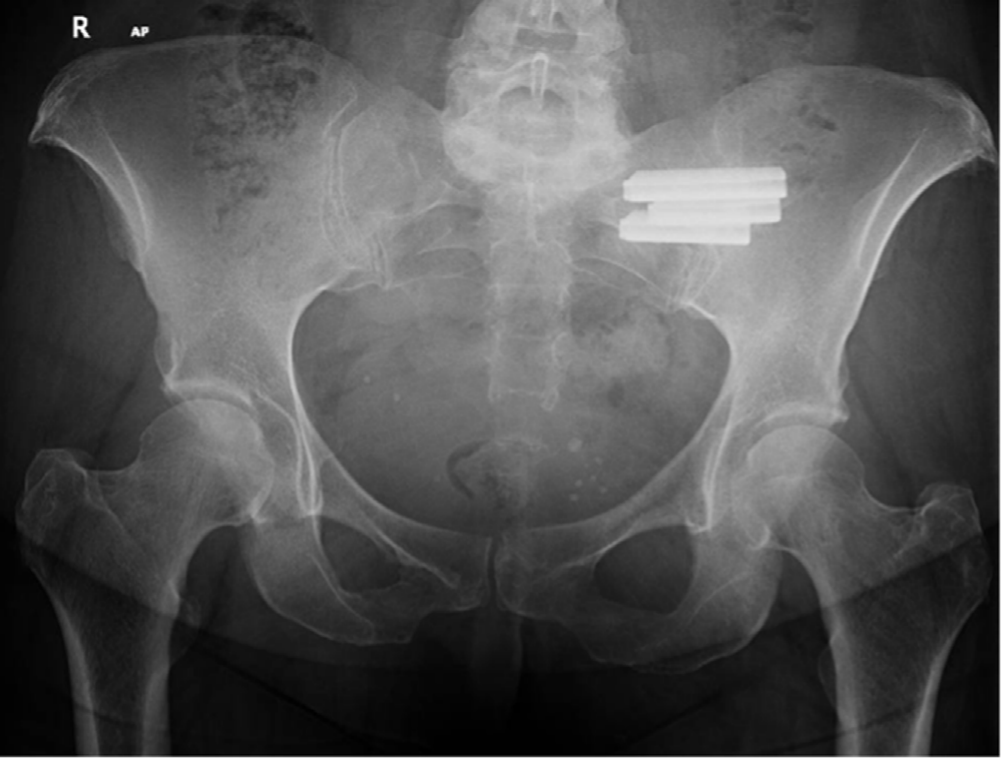
Ap X-ray of a pelvis, showing 1 year follow of left sacroiliac fusion, note absence of lysis or displacement, this was considered a radiological success.

A complication requiring a secondary procedure occurred in two patients (10%). Both patients developed radicular pain due to a breaching implant. One patient had an implant removal, and the other had a selective nerve root block (Figure 2).

An overall secondary procedure rate of 35% was observed. In addition to the aforementioned two patients, five patients (25%) presented with recurrent low back pain; one patient required an L4–L5 fusion, 3 required a facet injection, and one needed a secondary sacroiliac injection.

**Figure 3:**
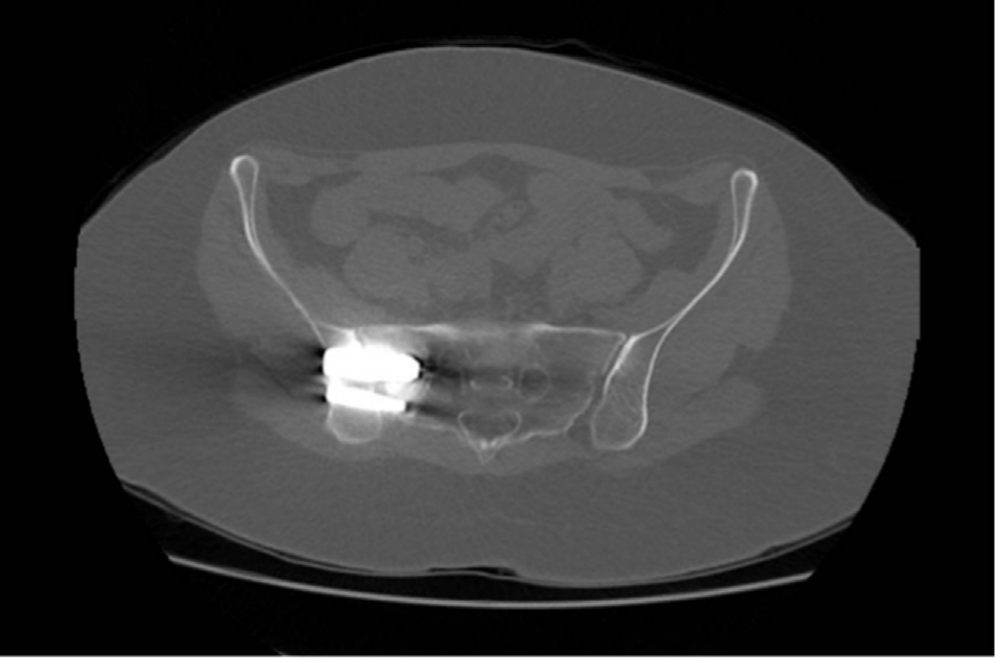
Axial cuts of a CT scan of pelvis, showing sacral osteolysis around caudal implants on the left side.

We found it difficult to confirm fusion on X-rays at the one-year mark; however, absence of significant displacement, implant breaches, or loosening was considered a radiological success (75% of patients) (Figure 2). Three patients had a screw breaching the bony corridors. Eight patients had a postoperative CT scan; 2 of them showed sacral osteolysis around the tip of the implant, and both had poor VAS and ODI scores at one year (Figure 3).

## Discussion

The sacroiliac joint is a well-recognized cause of low back pain [3]. Surgical management through minimally invasive fusion is rapidly emerging as the standard of care for resistant cases [7, 20]. We presented our results using the iFuse^®^ system with a minimum follow of 14 months. A statistically significant improvement in pain and functional outcomes was achieved, with only 55% and 50% achieving MCID using VAS and ODI, respectively. A 10% complication rate and a 35% secondary procedures rate were noted during the follow-up.

The study limitation is the small sample size and the lack of a comparative group. To our knowledge, this is the first study to report its outcomes achieved in day-to-day practice within the NHS setting with no conflict of interest.

The iFuse^®^ system for MI SIJ fusion is the most widely published method. There are two randomized controlled trials [2, 6], one multicentric prospective case series [21], three retrospective comparative studies [7–9], and 4 case series reporting their results using it [14, 16, 22, 23] (Table 1).

**Table 1:** Listing published work on MI SIJ fusion using iFuse^®^ implant.

Study	Years	Study	Number of patients undergoing iFuse procedure	Follow-up
Rudolf et al. [14]	2012	Case series	50	24
Cumming et al. [22]	2013	Case series	18	12
Gaetani et al. [23]	2013	Case series	12	8–18
Smith et al. [9]	2013	Retrospective multicenter comparative	114	24
Sachs et al. [16]	2014	Case series	144	16
Ledonio et al. [8]	2014	Retrospective comparative	27	13
Polly et al. [6]	2016	RCT	103	24
Duhon et al. [21]	2016	Multicentric case series	172	24
Vanaclocha et al. [7]	2017	Retrospective comparative	27	Min 12
Dengler et al. [2]	2019	RCT	53	24
This study	2022	Case series	20	19.9

This study’s diagnostic algorithm mirrors published studies (Table 1). All the studies relied on clinical examination and a positive response to a sacroiliac injection. This is based on criteria set by the International Association for the Study of Pain (IASP) [13]. These criteria are subjective and not 100% sensitive or specific [13, 24].

As the procedure is relatively new, it is unclear in the literature what the threshold is to offer fusion. In this study, conservative management was exhausted before offering surgery as a salvage procedure. A threshold of a VAS score of 50 and an ODI of 30 was used based on the work of Polly et al. In his trial, 84.4% of the conservative cohort failed conservative treatment and went on to undergo fusion after 6 months. This could serve as an estimate of how often fusion is required in cases crossing the threshold [6].

This study’s patient’s preoperative data are well matched to published studies with regard to age, sex, BMI, and functional status (Tables 2 and 3). A common feature is the high prevalence of previous lumbar surgery and the long duration of symptoms prior to surgery. An average preoperative ODI between 42 and 57.2 reflects the severe disability caused by SI pain (Table 3).

**Table 2:** Shows the demographics of this study’s patients compared to similar work.

Study	Mean age	Female %	Lumbar surgery %	Hip replacement/pathology %	Contra-lateral SI fusion %	Average BMI	Duration of symptoms/years
Rudolf et al. [14]	54	68	44	NA	0	NA	NA
Cumming et al. [22]	64	67	83	NA	0	31	NA
Gaetani et al. [23]	53	100					
Smith et al. [9]	57.4	71.9	47.4	NA	NA	NA	NA
Sachs et al. [16]	58	71	62	NA	NA	NA	NA
Ledonio et al. [8]	47.9	77.3	64	NA	NA	30.5	NA
Polly et al. [6]	50.2	73.5	40.2	15.7	NA	30.4	7.0
Duhon et al. [21]	50.9	69.8	44.2	14	0	29.4	5.1
Vanaclocha et al. [7]	48	70.4	7.4	3.7	NA	29.5	1.6
Dengler et al. [2]	49.4	73.1	34.6	NA	NA	26.5	4.9
This study	49.6	75	35	30	10	28.4	5.8

**Table 3:** List functional outcome reported on MI SI fusion using iFuse implant.

Study	Pain score preop	Mean improvement	% Achieving MCID	ODI preop	Change in ODI
Rudolf et al. [14]	76	49	82	–	–
Cumming et al. [22]	89	66	90	52.7	39.5
Gaetani et al. [23]	–	40	–	–	19.4
Smith et al. [9]	83	66	82	–	–
Sachs et al. [16]	86	59	90	–	–
Ledonio et al. [8]	–	–	–	54	12
Polly et al. [6]	82.3	55.6	83.1	57.2	28.5
Duhon et al. [21]	79.8	53.8	83.9	55.2	30.9
Vanaclocha et al. [7]	77.5	59.5	–	42	24
Dangler et al. [2]	77	45.5	79	58	26
This study	81.25	28.75	55	54.8	13.5

The mean improvement in functional scores and percentage of patients achieving MCID in this study is lower than the available literature (Table 3). This can be attributed to the diagnostic challenges, smaller population, and a higher complication rate. Twenty to thirty consecutive cases are needed to overcome complications in minimally invasive spine surgeries; whether the same learning curve applies to SI fusion is still to be proven [25]. Our operative cohort still had a more favorable outcome than the conservative cohorts in the Investigation of Sacroiliac Fusion Treatment (INSITE) and iFuse^®^ Minimally Invasive Arthrodesis (iMIA) trials [2, 6].

The iFuse^®^ implant design adds to the stability of fixation but decreases the margin of error during its insertion. Its cross-section is formed of an equilateral triangle, with each limb measuring 11.35 mm. This is substantially bigger than the biggest sacroiliac trauma screw at 8.0 mm. In this study, 10% of patients had an implant breech requiring a secondary procedure compared to 6% in the Rudolf et al. case series [14]. Dengler et al. had a higher rate of radiological breaches (32%), but only 1 case was symptomatic and required a secondary procedure [2]. Polly et al., Cummings et al., Smith et al., and Duhon et al. reported secondary procedures for implant-related nerve impingement requiring revision surgery [6, 9, 21, 22].

**Figure 4:**
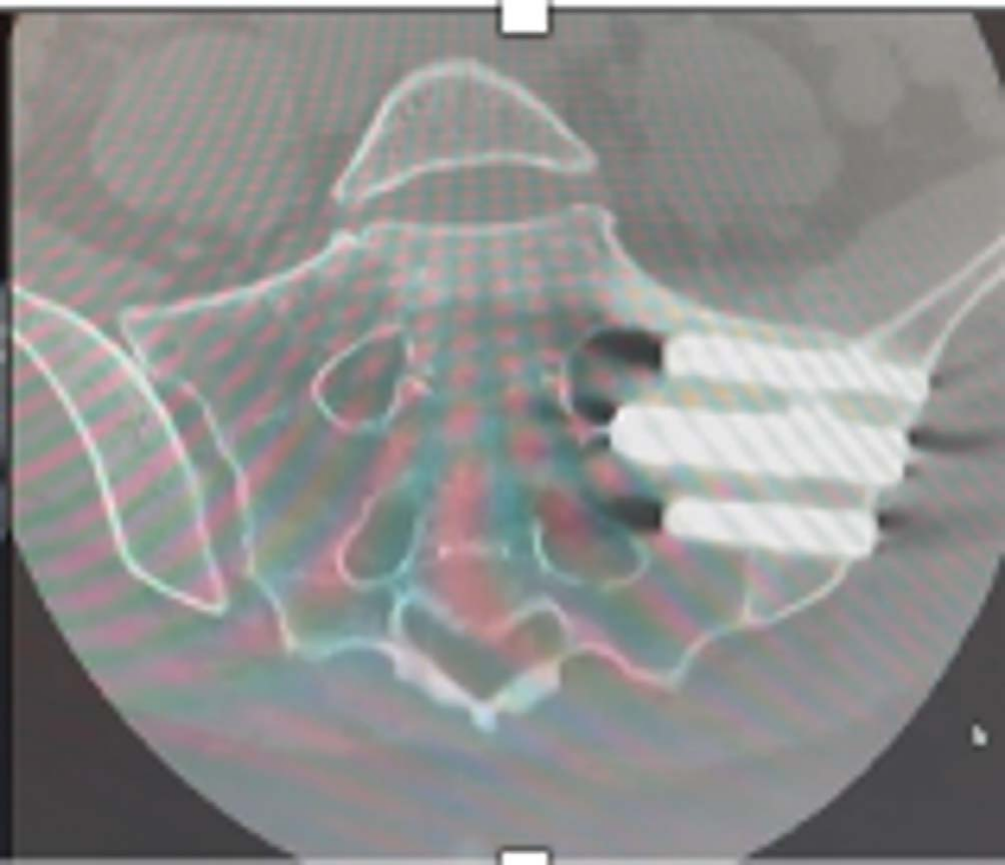
CT scan showing coronal cuts along axis of a dysmorphic sacrum. Note the high angulation of sacral alar and big neural foramina. The implant fixation was limited to Dennis I zone to allow for safe placement of implant.

The difficulty in instrumentation is further complicated by the sacral morphology. The dysmorphic sacrum bony corridor is more inclined superiorly in the frontal plane and anteriorly in the axial plane [26] (Figure 4). In this study, 40% of patients had at least one feature of dysmorphia, as described by Kaiser et al. [26]. The starting point and trajectory should be adjusted to match the plane of the SI corridor. Falzarno et al. reported the use of 3D navigation in iliosacral screw placement with low complications [27]. Polly et al. reported the use of 3d navigation in 2 centers in the INSITE trial, the safety of this subgroup had not been published separately [6].

Differentiation of SIJ pain from lumbosacral pathology proved challenging and can be a cause for secondary procedures. In this study, four patients (20%) represented after fusion with the pain believed to be from lumbosacral pathology. This is not unique to our study, Sachs et al. reported that 20% of his cohort represented facet joint pain [16]. It is difficult to determine whether these patients had a false positive response to the injection leading to an inaccurate diagnosis of SI as the main pain generator or whether these symptoms are due to secondary load transfer from the fused SI to the lumbar spine.

**Figure 5:**
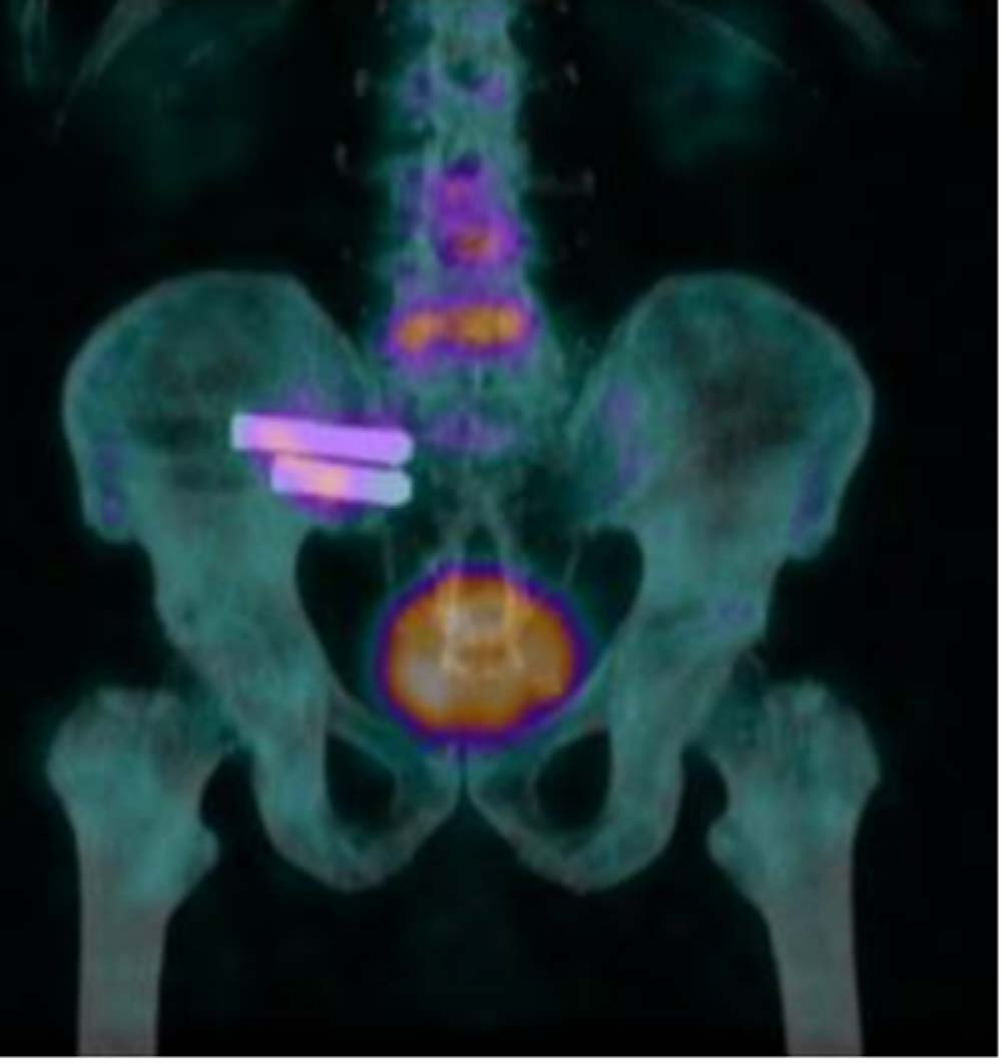
Coronal cut of a SPECT/CT showing cold right SI joint with metalwork insitu and a warm L4–L5 disc space. The patient later underwent L4–L5 fusion.

The lack of an imaging modality to confirm the diagnosis adds to the difficulty with standard cross-sectional studies being of limited utility [28]. Cusi et al. compared the single photon emission computed tomography (SPECT)/CT results to a clinical diagnosis of SI dysfunction based on provocative testing and reported 95% sensitivity and 99% sensitivity. In our study and all similar work, we have not used this modality as it is unvalidated against the response to sacroiliac injection, which is considered the gold standard [13]. We have used it selectively in patients with persistent symptoms after the procedure (Figure 5).

Radiological evaluation of fusion proved to be difficult on X-ray and CT scans. This has been echoed by Dengler et al. [2] and Duhon et al. [21]. The lack of implant breakage, migration, and lysis was considered a radiological success [2]. Sacral osteolysis noted on follow-up CT was also reported by Dengler et al. (15.1%). However, none of these patients had clinical signs attributed to that [2].

There is no consensus on the depth of implant insertion. Dengler et al. found no correlation between the length of implants and clinical results [2]. Duhon et al. reported on 17 implants with lysis, and almost all of them happened when there was less than 1 cm of the implant into the sacrum (21). In our study the length of the S1 implant was dictated by the safety of insertion. The narrowest diameter of the corridor is at the mid-alar area corresponding to Dennis zone II [26]. The fixation was limited to zone I when safety was in question (Figure 4).

No revisions were performed for the suspected failure of the fusion at the time of final follow-up. Duhon et al. reported 4 revisions for the suboptimal placement of implants [21]. Polly et al. and Dengler et al. had 1 revision each for suboptimal placement [2, 6]. No clear definition was given for what is considered suboptimal placement. The outcome of the revised patients has not been reported.

Our study sample size, heterogenicity of population, lack of comparative and high rate of complications did not allow for analysis of predictors of success. Dengler et al. performed a large meta analysis on two randomized controlled trials and a large multicentric prospective case series. The study reported better outcomes in nonsmokers, non-opioid users, older age groups, and longer pain duration [20].

Surgeons performing these procedures should be aware of the challenges with diagnosing the pathology and the challenges with safe implant positioning. Further work is needed to determine the utility of SPECT/CT in the primary diagnosis. Familiarity with instrumentation, surgical planning using cross-sectional imaging, and the use of 3d navigation could help increase the safety profile of the procedure.

In conclusion, the diagnosis and treatment of SI pain remains a difficult topic. Our results have shown that fusing SI joints is a difficult operation performed often in patients with chronic pain symptoms. However, all patients improved after SIJ fusion, some more so than others. There was a significant secondary procedure rate. We believe that most patients who did poorly fell into one of three groups, wrong diagnosis, a complication, or another condition that resulted in a poor outcome, such as failed back syndrome. Further work is needed to help identify patients who will have the best results from this surgery.

## Conflict of interest

MHA certifies that he has no financial conflict of interest. WA certifies that he has no financial conflict of interest. SAK certifies that he has no financial conflict of interest. KFMA certifies that he has no financial conflict of interest. MAC certifies that he has no financial conflict of interest. JG certifies that he has no financial conflict of interest. PMS certifies that he has no financial conflict of interest.

## Funding

The research did not receive any specific funding.

## Ethical approval

Ethical approval was not required.

## Informed consent

Written informed consent was obtained from all patients for the procedure, and oral consent for the collection of functional scores and use of their data.

## Author contribution

MHA data collection, writing up of original draft, and analysis. WA writing up, editing, and analysis. SAK reviewing and editing. KFMA supervision and conceptualization. MAC clinical contribution. JG clinical contribution. PMS conceptualization, supervision, clinical contribution, reviewing, and editing.
